# Rifabutin-associated hypopyon uveitis and retinal vasculitis with a history of acute myeloid leukemia

**DOI:** 10.1007/s12348-012-0059-9

**Published:** 2012-02-04

**Authors:** Wendy M. Smith, Madhu G. Reddy, Kelly A. Hutcheson, Rachel J. Bishop, H. Nida Sen

**Affiliations:** 1 Laboratory of Immunology, National Eye Institute, National Institutes of Health, 10 Center Dr., Building 10, Rm. 10N112, Bethesda, MD 20892 USA; 2 Consult Services Section, National Eye Institute, National Institutes of Health, 10 Center Dr., Building 10, Rm. 10C432A, Bethesda, MD 20814 USA; 3 Department of Ophthalmology, Children’s National Medical Center, Washington, DC USA

**Keywords:** Uveitis, Rifabutin, Retinal vasculitis, Cystoid macular edema

## Abstract

**Purpose:**

This study reports a case of bilateral rifabutin-associated uveitis in a child with a history of acute myeloid leukemia.

**Methods:**

We utilized a clinical case description and brief discussion.

**Results:**

A 17-year-old girl presented with acute bilateral anterior uveitis, a hypopyon in the left eye, and moderate bilateral vitritis. She had a history of acute myeloid leukemia status post-allogeneic hematopoietic stem cell transplant 5 years earlier. She was receiving rifabutin for a biopsy-proven *Mycobacterium avium* complex pulmonary infection. Work up for infectious and neoplastic etiologies was negative. The uveitis initially responded to topical corticosteroids, but recurred when the drops were tapered. Fluorescein angiography demonstrated diffuse vasculitis of small retinal vessels and cystoid macular edema. After rifabutin was discontinued, the uveitis and vasculitis slowly resolved.

**Conclusion:**

Fluorescein angiography demonstrated widespread retinal vasculitis which is a rare manifestation of rifabutin-associated uveitis.

## Introduction

Rifabutin is a semisynthetic derivative of rifampin that is effective for the prophylaxis and treatment of *Mycobacterium avium* complex (MAC) infection. It is frequently used in immunocompromised patients with human immunodeficiency virus (HIV) or a history of organ or stem cell transplantation. Anterior uveitis and hypopyon are well-recognized and frequently reported complications of rifabutin treatment; however, posterior involvement is relatively rare [1, 2]. Here, we describe a case of rifabutin-associated panuveitis in a young patient with a history of acute myeloid leukemia.

## Case report

A 17-year-old Eritrean girl presented with a 5-day history of redness, tearing, and photophobia which started in her right eye (OD) and then involved her left (OS). She recalled similar, but less severe symptoms over the 2 months prior to presentation. Her medical history was significant for acute myeloid leukemia diagnosed 5 years earlier. She was treated with chemotherapy and received an allogeneic hematopoietic stem cell transplant 6 months after diagnosis. She developed graft-versus-host-disease (GVHD) of the gastrointestinal tract, lungs, and skin with mild ocular surface involvement, but these issues were stable at the time of presentation to the eye clinic.

**Fig. 1:**
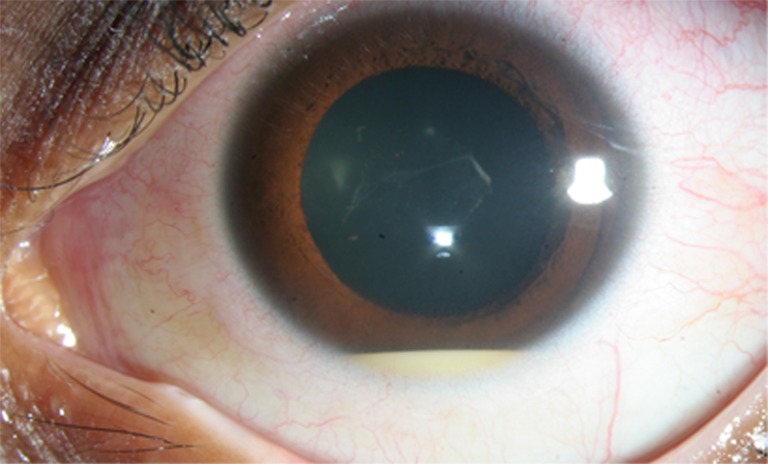
Anterior segment photograph of the left eye showing a small hypopyon and visible vitreous debris (posterior synechiae were broken by dilation)

On initial examination, pinhole visual acuity was 20/32 in each eye (OU), and intraocular pressures were 14 mmHg OU. Small non-granulomatous keratic precipitates were present OU. The anterior chambers had 3+ and 4+ cells (OD and OS, respectively), and bilateral moderate vitritis was noted. The left eye also had a hypopyon (less than 1 mm) and posterior synechiae of the iris (Fig. 1). Both eyes were treated with topical prednisolone acetate 1% every hour and cyclopentolate twice daily. Due to the concern for recurrent leukemia or infection, aqueous humor was obtained from the left eye; cytology was negative for malignant cells, and cultures were negative. Laboratory testing was negative or within normal limits for RPR, syphilis IgG, hepatitis B and C, angiotensin-converting enzyme, lysozyme, ANA, anti-dsDNA, rheumatoid factor, and HLA-B27. The uveitis resolved after 2 weeks of intensive topical corticosteroids.

**Fig. 2:**
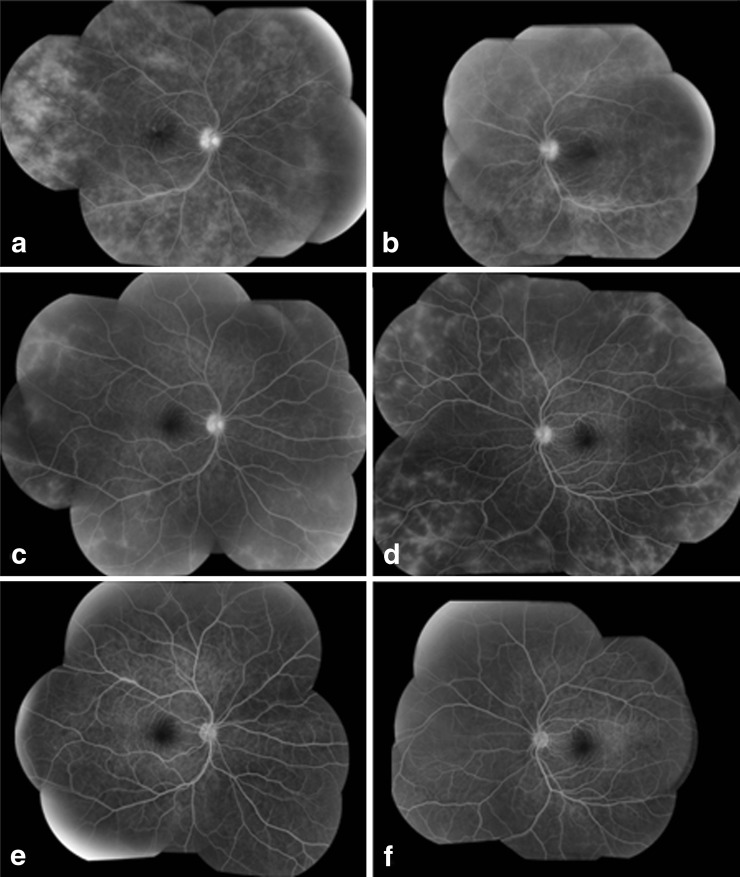
Fluorescein angiography montages of the right and left eyes: **a**, **b** Baseline (prior to discontinuation of rifabutin). **c**, **d** Five weeks after stopping rifabutin. **e**, **f** Four months after stopping rifabutin

**Fig. 3:**
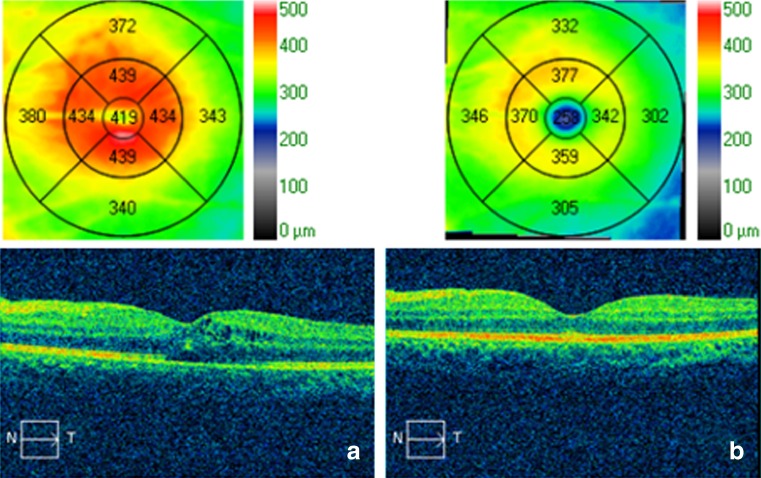
Cirrus OCT of the left eye: Cystoid macular edema present 9 days (**a**) after stopping rifabutin that resolved 1 month later (**b**)

Topical corticosteroids were tapered to every 2 hours OU. Ten days later, the patient developed recurrent bilateral anterior uveitis and vitritis with a hypopyon OS. Fluorescein angiography (FA) showed diffuse retinal vascular leakage and late staining of the optic nerves in both eyes (Fig. 2a, b). A more detailed review of her medications and medical history revealed she was on rifabutin for recurrent MAC. Nearly 2 years before she developed uveitis, she had completed a 9-month course of ethambutol, clarithromycin, and rifabutin for MAC cultured from a lung biopsy. During the same period, she was on systemic immunosuppressive medications (mycophenolate mofetil, cyclosporine, and prednisone) for GVHD. Eight months before presenting with hypopyon uveitis, ethambutol, clarithromycin, and rifabutin were restarted for recurrent MAC. Rifabutin dosing was twice as high (300 mg every Monday, Wednesday, and Friday), and she was no longer on systemic immunosuppressive agents. After rifabutin-associated uveitis was diagnosed, her medication was switched from rifabutin to moxifloxacin. Topical corticosteroids were continued at the same frequency (every 2 hours), and the hypopyon resolved 5 days later. In the first 2 weeks of follow-up, the macular edema initially worsened, OS > OD, but over time, the thickening gradually returned to normal (Fig. 3). On fluorescein angiography, the diffuse retinal leakage had improved at 5 weeks and had resolved after 4 months on a slow topical corticosteroid taper (Fig. 2c–f).

## Discussion

Anterior uveitis and hypopyon are the most frequent manifestations of rifabutin-associated uveitis; however, severe cases may develop dense vitritis with large yellow-white opacities or panuveitis resembling endophthalmitis [3, 4]. Despite the strong association with rifabutin, infectious, autoimmune and neoplastic etiologies should still be investigated depending on the clinical situation. Examination of aqueous humor and/or vitreous via culture, cytology, or polymerase chain reaction can help rule out infection or malignancy.

Although mild to moderate vitritis frequently develops in rifabutin-associated uveitis, posterior involvement is less frequent [5–7]. Similar to the previous cases, our patient presented with uveitis several months after the start of rifabutin and responded quickly to topical corticosteroids and discontinuation of rifabutin. Of the four cases described by Skolik, only one patient had diffuse vasculitis on FA; the others had mild peripheral vasculitis or perivascular sheathing. Of note, the patient with diffuse FA changes was immunocompetent, whereas the other patients had acquired immunodeficiency disease syndrome (AIDS) and CD4 counts less than 100 cells/mm^3^. In each of the single case reports by Arevalo and Vaudaux, the rifabutin-associated uveitis was unilateral and the posterior findings were focal. Arevalo’s patient had AIDS and a CD4 count of 3 cells/mm^3^; a focal area of sclerosed retinal vessels was noted after intraocular injections of vancomycin and gentamicin for presumed endophthalmitis. In the case from Vaudaux, the patient had been treated with systemic prednisone for chronic obstructive pulmonary disease 4 months prior to developing uveitis, but was otherwise immunocompetent. The FA and ocular coherence tomography (OCT) confirmed unilateral cystoid macular edema. In our immunocompetent patient, the vasculitis was notable for its diffuse and bilateral nature and slow resolution after the discontinuation of rifabutin.

Rifabutin-associated uveitis was initially described in HIV-positive patients, but it may also occur in patients who are immunocompetent [7]. Only a few pediatric cases have been reported [8]; however, due to the possible association with low body weight [2], weight-based dosing has been suggested as a strategy to prevent uveitic complications. Our patient weighed 42.5 kg, and uveitis developed after the rifabutin was restarted at twice the initial dose. It is unclear whether the lack of systemic immunomodulatory medications influenced the onset of uveitis during the second course of rifabutin since patients have developed rifabutin uveitis while iatrogenically immunosuppressed after transplantation [9]. Perhaps an intact immune system predisposed our patient to develop more extensive retinal vasculitic changes.

## Conclusion

We describe a case of rifabutin-associated hypopyon uveitis in a young immunocompetent patient with an unusual degree of diffuse bilateral retinal vasculitis. While the anterior chamber and vitreous inflammation disappeared rapidly after discontinuation of rifabutin, resolution of the retinal vasculitis took several months. Fluorescein angiography helped assess the extent and severity of uveitis as well as guide the topical corticosteroid taper.
